# Pain relief by touch: A quantitative approach

**DOI:** 10.1016/j.pain.2013.12.024

**Published:** 2014-03

**Authors:** Flavia Mancini, Thomas Nash, Gian Domenico Iannetti, Patrick Haggard

**Affiliations:** a Institute of Cognitive Neuroscience, University College London, London, UK; b Department of Neuroscience, Physiology and Pharmacology, University College London, London, UK; c Division of Medicine, University College London, London, UK

**Keywords:** Pain, Touch, Relief, Analgesia, Space, Signal detection theory

## Abstract

Pain relief by touch has been studied for decades in pain neuroscience. Human perceptual studies revealed analgesic effects of segmental tactile stimulation, as compared to extrasegmental touch. However, the spatial organisation of touch–pain interactions within a single human dermatome has not been investigated yet. In 2 experiments we tested *whether*, *how*, and *where* within a dermatome touch modulates the perception of laser-evoked pain. We measured pain perception using intensity ratings, qualitative descriptors, and signal detection measures of sensitivity and response bias. Touch concurrent with laser pulses produced a significant analgesia, and reduced the *sensitivity* in detecting the energy of laser stimulation, implying a functional loss of information within the ascending Aδ pathway. Touch also produced a bias to judge laser stimuli as less painful. This bias decreased linearly when the distance between the laser and tactile stimuli increased. Thus, our study provides evidence for a spatial organisation of intrasegmental touch–pain interactions.

## Summary

We tested *whether*, *how*, and *where* intrasegmental touch modulates the perception of laser-evoked, acute pain. We provide evidence for a spatial organisation of touch–pain interactions within a single dermatome.

## 1 Introduction

Pain relief by touch has been central to the study of pain mechanisms [19,28]. Neurophysiological investigations in animals indicate a class of wide dynamic range (WDR) neurons in the dorsal horn as a likely substrate of the analgesia induced by touch [14]. These neurons are multimodal, in that they respond to both nociceptive and tactile inputs. The structure of their receptive field (RF) is characterised by an excitatory centre and an inhibitory surround. Studies of WDR neurons in animals have shown a spatial gradient of inhibition: an intense tactile stimulus in the periphery of the inhibitory field could reduce the response to a nociceptive stimulus as much as a less intense tactile stimulus farther from the periphery [8,25,26].

However, RFs and firing rates of spinal neurons cannot be readily measured in humans. Instead, the spatial pattern of interactions between large and small fibres has been considered categorically, using a binary RF model. Many human studies contrasted the effect of segmental vs extrasegmental stimulation on pain thresholds or perceived pain levels [7,13,22,23,29,30]. These studies found that tactile inputs inhibited pain perception segmentally, but not when tactile and nociceptive inputs were delivered to different dermatomes.

The spatial field of multisensory interactions between cutaneous inputs can also be considered in a continuous way, by varying the distance between tactile and nociceptive stimulation within a single dermatome, and investigating the spatial dependency of touch–pain interactions. To our knowledge, the segmental spatial organisation of tactile influence on pain perception has not been systematically studied in humans.

In 2 experiments, we tested *whether*, *how*, and *where* spatiotemporally defined tactile input modulates the perception of laser-evoked pain in healthy volunteers. Our study aimed to go beyond previous investigations of these questions on humans, by combining for the first time nociceptive-selective stimulation with signal detection theory (SDT) [15] to study the spatial dependency of touch–pain interactions within a single dermatome.

## 2 Experiment 1

In Experiment 1, we investigated which aspects of pain perception are modulated by non-nociceptive somatosensory stimulation using von Frey filaments: intensity level, quality of percept, latency of detection. We also investigated tactile effects of sensitivity and response bias in judging pain levels, using SDT. Although SDT has been used previously in pain research [24], the majority of studies tested pain detection (“Does the stimulus cause pricking pain, yes or no?”), rather than level detection within the pain range (“Is the pain level high or less high?” [17]). The first approach focuses on whether the Aδ pathway is activated or not. The second approach focuses instead on information *within* the ascending Aδ pathway, and may be more relevant to perception of pain *level*.

### 2.1 Method

#### 2.1.1 Participants

Eight healthy volunteers (5 females) aged 19-32 years (mean ± SD, 22 ± 4.0 years) participated for payment. Four participants were right-handed, and four were left-handed. All participants were recruited through a departmental subject pool, and gave written informed consent to take part in the experiment. Experimental procedures were approved by the University College London ethics committee (approval number: 3167/001). Eligibility criteria included: 1) no history of neurological disorders; 2) not having taken any analgesic medications nor recreational drugs in the 24 hours preceding the experiment; 3) not having participated in a brain stimulation study in the 24 hours preceding the experiment; 4) white skin, because at the wavelength of the neodymium:yttrium-aluminium-perovskite (Nd:YAP) laser we used, the radiation absorption of dark skin is larger [1].

#### 2.1.2 Laser stimuli

We delivered pulses of radiant heat generated by an infrared Nd:YAP laser with a wavelength of 1.34 μm (EIEn, Florence, Italy). This method was used to selectively activate nociceptive Aδ and C-fibre endings located in the superficial layers of the hairy skin [1]. The laser pulse was transmitted via an optic fibre, and focused by lenses to a spot diameter of approximately 3.5 mm. A visible He-Ne laser spot was used to point the Nd:YAP laser to the target location. The duration of each laser pulse was 4 ms.

**Fig. 1:**
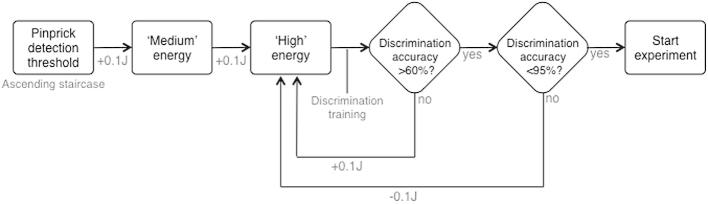
Selection of experimental laser energies*.* The pinprick detection threshold was determined for each subject, and the “medium” energy level was set as +0.1 J above this level. The energy of the “high” stimulus was then adjusted in 2 ways: 1) Increasing steps of +0.1 J, until the accuracy of discriminating between the 2 levels was >60%; 2) decreasing steps of −0.1 J, until the accuracy of discriminating between the 2 levels was <95%.

For each participant, we identified the pinprick detection threshold using ascending staircases (mean ± SE, 0.39 ± 0.002 J). The threshold was identified as the first stimulus energy that elicited reports of pinprick sensation for 3 consecutive repetitions. We then set 2 suprathreshold laser energies for the main experiment (“medium” and “high”; Fig. 1). The “medium” energy was set as 0.1 J above individual detection threshold (mean ± SE, 0.49 ± 0.002 J). The “high” energy was initially set as 0.2 J above individual detection threshold. Next, we verified that participants could distinguish between these levels by asking participants to respond whether 20 stimuli presented at random were medium or high. If the discrimination accuracy was >60% and <95%, the experiment started with these levels. If the discrimination accuracy was <60%, the energy of the “high” stimulus was increased in steps of 0.1 J until the minimum accuracy of 60% was reached. If the discrimination accuracy was >95%, the energy of the “high” stimulus was decreased in steps of 0.1 J until the maximum accuracy of 95% was reached (Fig. 1; mean energy of high stimulus ± SE, 0.62 ± 0.004 J). The mean accuracy of discriminating the stimuli presented in the experiment was 77.3% (SE ± 2.86%). Four additional participants were unable to discriminate the 2 energy levels according to this procedure, and therefore were not tested in any session.

**Fig. 2:**
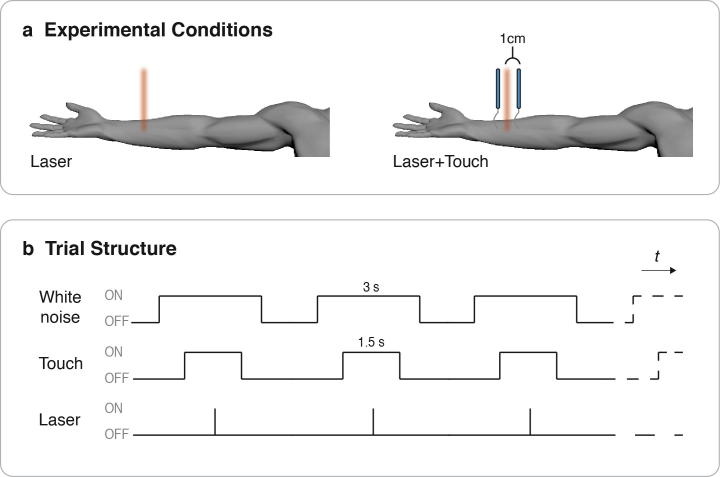
(a) Experimental conditions. In Experiment 1, laser pulses were given to the volar skin of the left forearm (“Laser” condition). In the “Laser + Touch” condition, 2 von Frey hair filaments accompanied the laser pulse, 1 cm either side. (b) Trial structure. All stimuli were delivered in the centre of a 3-second burst of white noise. Laser pulse duration was 4 ms. In “Laser + Touch” trials, tactile stimulation was applied for 1.5 seconds bracketing the laser pulse.

In order to avoid receptor fatigue or sensitisation, the stimulation was alternated across 4 different skin locations along the radial–ulnar axis of the left ventral forearm, approximately halfway between the elbow and the wrist (Fig. 2a). Since the output energy depends on the skin temperature, we monitored the temperature of the stimulated surface with an infrared thermometer every 16 trials. Average skin temperature was 32.6°C (SE ± 0.12°C).

#### 2.1.3 Tactile stimuli

Tactile stimuli consisted of a pair of calibrated nylon filaments (von Frey hair, 1 g, diameter 0.4 mm) mounted 2 cm apart. The tactile stimuli were applied to the skin for 1.5 seconds by a computer-controlled 3-axis robot (Arrick Robotics, Tyler, TX, USA). Robotic positioning ensured that the tactile stimuli bracketed the site of laser stimulation, so that the distance between laser stimulation and each of the pair of tactile inputs was exactly 1 cm (Fig. 2a). Note that the tactile stimulation includes both a dynamic and a static component, and is likely to stimulate both fast- and slow-adapting somatosensory afferents [11].

#### 2.1.4 Experimental procedure

Participants sat comfortably with their left forearm lying outstretched. They were blindfolded, and wore headphones. Every participant took part in 2 separate sessions in counterbalanced order. The same experimental conditions were given in the 2 sessions: 2 laser energies were used (“medium” and “high”). Importantly, both energies were above the pinprick pain threshold (see above). On each trial, the laser pulse could either be applied alone (Laser condition, L), or together with a pair of von Frey hair filaments (Laser + Touch, L+T; Fig. 2a-b).

We collected different measures in the 2 sessions. In session A, the pain level evoked by each laser pulse was reported as “high” or “not high” in a forced-choice paradigm. SDT was used to obtain independent estimates of perceptual sensitivity and response bias. In session B, the same participants provided reaction times, ratings of subjective pain intensity, and verbal descriptors of the quality of the percept.

In both sessions (Fig. 2b), white noise was played on every trial, from 1.5 seconds before the onset of the laser stimulation to 1.5 seconds after. This provided an auditory cue for the following stimulation, controlling for any possible cueing effect introduced by the tactile stimulation. It also masked the noise made by the robot movement. In the Laser + Touch condition, touch was applied 0.75 second before the laser onset, and lasted 1.5 seconds. The experimental condition (Modality: L, L+T; Energy: medium, high) was randomised. The experimenter (T.N.) was blinded to both the tactile and the laser stimulation settings. The experiment lasted around 90 minutes. In session “A” (forced-choice paradigm), 10 blocks consisting of 4 trials each were presented. In session “B,” we introduced an additional 20% of catch trials in which no laser stimulus was given; each condition was repeated 10 times.

#### 2.1.5 Measures and analyses

In session “A,” participants were required to discriminate the energy of a single laser stimulus, whether it was “high” or not. Note that the participants were not asked to detect pain (since all stimuli were set to elicit pricking pain), but to discriminate between a more or less intense level of stimulation. This paradigm allowed us to separately assess sensitivity and response bias according to SDT [15].

A corollary of this design is that performance depends on memory, since participants must first learn and then remember what the high and low energy stimuli feel like. However, many perceptual paradigms involve a memory element. Even the classic 2-interval, 2-alternative forced-choice paradigm involves remembering the first stimulus of a pair until the second can be compared to it. Although such a recalled representation of the signal is involved in many psychophysical paradigms, this should equally affect all the experimental conditions.

We calculated normalised hit rates (*P*[“high”/high-energy stimulus], proportion of hit trials to which subject responded “high”), and false alarm rates (*P*[“high”/medium-energy stimulus], proportion of trials in which the laser energy was medium but the subject responded “high”). These were used to obtain the perceptual sensitivity (*d*′), a measure of discriminability in detecting the high-intensity target, and the response bias (*C*), which measures the tendency to report stimuli as “high.” The sensitivity (*d*’) was quantified as: *d*’ = z(hit rate) − z(false alarm rate). The response bias (*C*) can be expressed as: *C* = (z[hit rate] + z[false alarm rate]) * 0.05. Sensitivity and response bias values were calculated for each modality (L, L+T).

In session “B,” in each trial participants were required to 1) press a key as soon as they detected the laser pulse; 2) rate the subjective intensity of the pain on a scale of 0-100 (0 = no pain, 100 = worst pain imaginable); 3) describe the quality of their sensation by choosing one of the following 6 verbal descriptors: “pricking,” “warm,” “dull,” “pressing,” “tingling,” “none.” These descriptors were chosen on the basis of a previous study that identified them among 77 words as being best able to differentiate between Aδ and C-fibre pain [2]. Trials in which the laser stimulus was not perceived at all by the participant (no key press recorded and chosen descriptor “none”: “imperceptions”) were analysed separately from trials in which the laser stimulus was perceived.

### 2.2 Results

#### 2.2.1 Sensitivity (*d*’)

**Fig. 3:**
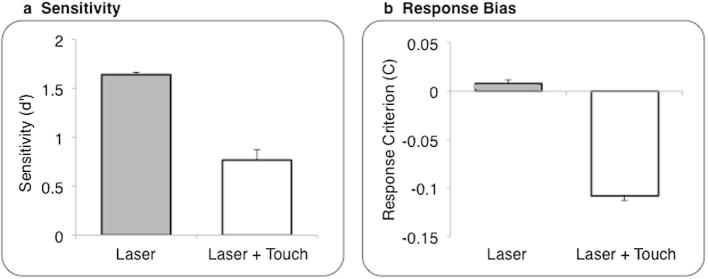
Experiment 1: signal detection results*.* Group average (± SE) sensitivity (a) and response bias (b) measures for each modality of stimulation; n = 8.

Sensitivity was significantly reduced when laser stimuli were accompanied by concurrent tactile stimulation (L+T) as compared to the baseline L condition (Fig. 3a; paired *t*-test: *t*_(7) _= 2.58, *P* = 0.037).

#### 2.2.2 Response bias (C)

We observed a highly significant reduction in response bias in the L+T condition as compared to the baseline L condition (Fig. 3b, paired *t*-test: *t*_(7) _= 6.37, *P* < 0.0001). A negative response bias indicates a tendency to report “medium.” Therefore, concurrent touch produced a general shift to report lower pain levels, independent of the actual laser energy delivered.

#### 2.2.3 Probability of detection

**Fig. 4:**
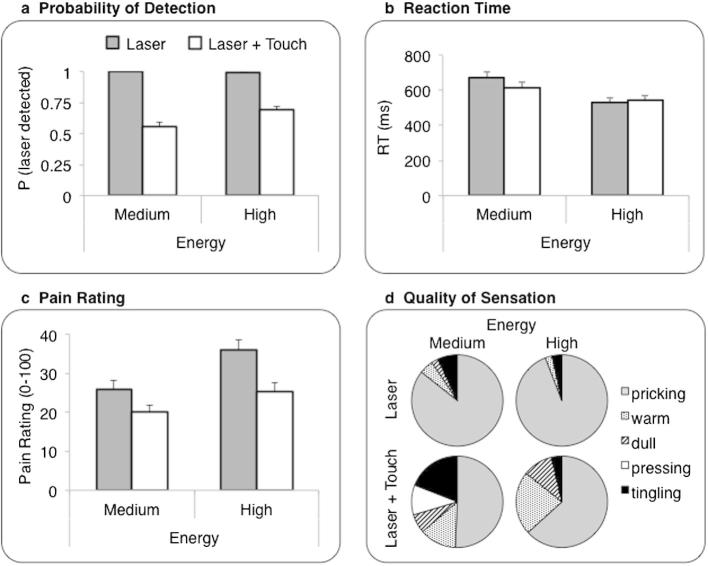
Experiment 1: (a) probability of detection, (b) reaction time, (c) rating of subjective pain intensity. Group average (± SE) values for each modality of stimulation (Laser, Laser + Touch) and laser energy (medium, high). (d) Quality of sensation. The mean proportion of agreement with each descriptor is plotted in each condition. Only trials in which the laser stimulus was detected are included; n = 8.

In the baseline (L) condition of session “B,” the probability of detection, based on the key-press response and “none” quality of sensation, was 98.9% (SE ± 1.1%) for high-energy laser stimuli. One hundred percent (SE = 0) of medium energy stimuli were detected. The probability of detection of laser stimuli was dramatically affected by concurrent touch (L+T). Detection levels dropped to 69.2% (SE ± 7.2%) in high-energy L+T trials and to 56% (SE ± 8%) in medium-energy L+T trials (Fig. 4a). A 2 × 2 repeated-measures analysis of variance on the probability of laser pulse detection, with factors of stimulus modality (L, L+LT) and stimulus energy (medium, high) revealed a highly significant main effect of modality [*F*(1, 7) = 30.7 *P* = 0.001]. The main effect of energy was not significant [F(1, 7) = 2.51, *P* = 0.157], though there was a trend toward an interaction [F(1, 7) = 4.16, *P* = 0.081]. Trials in which the laser was not detected were not included in the subsequent analyses.

#### 2.2.4 Reaction time

Reaction times (RTs) for trials in which the laser stimulus was detected (Fig. 4b) were within the Aδ conduction range [ie, <650 ms, 20]. Importantly, RTs were not affected by the presence of concurrent touch (F < 1). There was an almost significant main effect of laser energy [F(1, 7) = 5.42, *P* = 0.053], with faster RTs to high-energy than to medium-energy laser stimuli. There was no significant interaction (F < 1), suggesting that the effect of stimulus energy was independent of modality.

#### 2.2.5 Rating of pain intensity

Pain ratings showed the expected positive relation with laser energy [F(1, 7) = 9.89, *P* = 0.016]. Concurrent touch (L+T) significantly reduced pain ratings as compared to the baseline (L) condition [F(1, 7) = 14.30, *P* = 0.007]. This tactile analgesia effect did not interact with energy level [F(1, 7) = 3.29, *P* = 0.112; Fig. 4c].

#### 2.2.6 Quality of sensation

The probability of choosing each of the 6 available descriptors was recorded for those trials in which the laser stimulus was detected. The results are shown in Fig. 4d. In the baseline (L) condition, 93% and 86% of high- and medium-energy trials, respectively, were described as “pricking.” In trials with concurrent tactile stimulation (L+T), only 42% and 28% of high- and medium-energy trials, respectively, were described as “pricking.” Importantly, the RTs in those L+T trials described as “warm” and “dull” (mean ± SE, 616 ± 73.4 ms) did not differ significantly from the RTs for those trials described as “pricking” (mean ± SE, 541.6 ± 69.8 ms; paired *t*-test: *t*_(7) _= −0.96, *P* = 0.367). Thus, even when pricking quality was not the primary reported sensation on L+T trials, the conduction velocities were still in the Aδ range [20].

#### 2.2.7 Multivariate analyses of tactile modulation

We conducted a linear discriminant analysis on the trials that were detected to investigate what linear combination of our dependent measures best captured the effects of touch on pain perception. We included the following measures: 1) average raw pain ratings for medium and high energy stimulation, 2) difference in pain ratings between medium and high energy, 3) response bias in reporting laser energy, 4) sensitivity in discriminating laser energy. The overall discriminant model was significant [Wilks’ Lambda = 0.031234, approximated by F(4, 4) = 31.02, *P* = 0.003]. The standardised weighting coefficients in Table 1 show how much each dependent variable contributed to the modulatory effects of touch. SDT response bias was the major contributor to the modulatory effect of touch, closely followed by average pain ratings. Energy-related differences in pain ratings were less effective in separating trials with and without concurrent touch, and the SDT sensitivity measure (*d*’) was even less effective. In other words, response bias in a signal detection task is the most prominent sensory aspect of tactile analgesia, closely followed by pain ratings. In contrast, tactile stimulation had a comparatively modest effect on perceptual information processing in the nociceptive system, as measured by SDT.

### 2.3 Discussion

Experiment 1 demonstrated that tonic touch concurrent with Aδ laser stimulation produced a significant analgesia, consisting in decreased sensitivity and negative bias in a signal detection task, reduced probability of detecting the laser stimulus, and decreased ratings of pain intensity. Interestingly, touch also influenced the quality of sensation. Laser stimulation was not primarily described as “pricking” when touch was co-applied, although reaction times indicated that these stimuli still activated Aδ pathways, according to the conventional criterion [20].

## 3 Experiment 2

Experiment 2 investigated where, within a dermatome, touch relieves pain, by varying the spatial relation between tactile and laser stimulation, and measuring sensitivity and response bias. In Experiment 1, touch was applied 1 cm either side of the laser pulse on the forearm (1 cm on both sides, Fig. 2a). In Experiment 2, we applied similar tactile stimulation at 1, 5, and 9 cm either side of the laser pulse, and used signal detection measures to assess the effects of touch location on pain.

### 3.1 Method

#### 3.1.1 Participants

Fourteen healthy volunteers (8 females) aged 18-28 years (mean ± SD, 23 ± 3.3 years) participated for payment. One participant was left-handed. The recruitment procedures, eligibility criteria, and the ethical approval were identical to Experiment 1.

#### 3.1.2 Stimuli

The laser stimuli were identical to Experiment 1. Tactile stimuli were as in Experiment 1, but were positioned 1, 5, and 9 cm symmetrically either side of the laser beam, along the proximal-distal axis of the forearm. A no-touch baseline condition (L) was also included, as in Experiment 1. The laser energies for the medium (mean ± SE, 0.5 ± 0.001 J) and high stimulus (mean ± SE, 0.68 ± 0.001 J) were selected following the same procedure of Experiment 1. Three additional participants did not reach the inclusion criterion (<60% discrimination accuracy), and therefore were not tested.

Detection thresholds were, on average, 0.41 J (SE ± 0.001 J). Reaction times to detection of laser pulses were evaluated before the experiment to ensure that both energies did indeed activate Aδ fibres. Average values for both “high” and “medium” energies (High: mean ± SE, 456 ± 6.1 ms; medium: 579.6 ± 5.8 ms) were compatible with the conduction velocity of Aδ fibres [20].

#### 3.1.3 Procedure

The same paradigm as session “A” of Experiment 1 was used. Participants judged whether laser energy was “medium” or “high.” Three L+T conditions (1, 5, 9 cm) and one L condition at 2 energy levels were randomised. Ten blocks of 8 trials were presented, interrupted by short rest breaks, for a total of 80 trials. The experiment lasted around 90 minutes.

#### 3.1.4 Analyses

Sensitivity (*d*’) and response bias (*C*) were calculated as before, separately for each condition (L, L+T9, L+T5, L+T1). The results were analysed using 2 planned contrasts and a set of post hoc tests, applied to each measure (*d’* and *C*). The first contrast tested the hypothesis that intrasegmental touch modulated *d’* and *C*. This contrast compared the L to the average of all the L+T conditions (ie, weights −1, 1/3, 1/3, 1/3). This contrast was designed to replicate the effects of Experiment 1, but also to extend them across a wider spatial separation, with up to 9 cm between laser and tactile stimulation. The second contrast was designed to investigate the spatial organisation of the touch–pain interaction within this zone. We hypothesised a linear relation between spatial location of touch and SDT measures, based on the principle of spatially graded multisensory interactions [8,25,26]. To test this hypothesis, we used a linear trend contrast, with weights −1, 0, and 1 for the 3 tactile conditions L+T9, L+T5, and L+T1, respectively [6].

Finally, we also performed 2-tailed Dunnett’s tests on each SDT measure, to compare each of the 3 L+T conditions against the baseline (L). Dunnett’s test, which controls the type I error rate when comparing several means to a single control, allowed us to identify whether touch at each spatial location significantly modulated pain or not.

### 3.2 Results

#### 3.2.1 Sensitivity (*d*’)

**Fig. 5:**
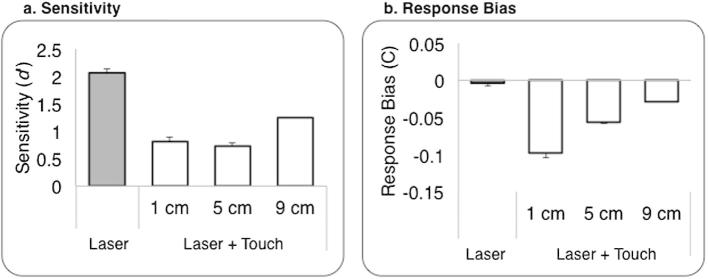
Experiment 2: (a) sensitivity and (b) response bias results. Group average (±SE) sensitivity (*d*’) and criterion (*C*) scores were plotted by the experimental condition (Laser, Laser + Touch: 1, 5, 9 cm either side of the laser pulse); n = 14.

The first contrast (L vs average L+T) showed that, overall, touch on the volar skin of the forearm significantly decreased *d*’ (*P* < 0.0001), as shown in Fig. 5a. The contrast for the linear trend (*P* = 0.239) did not identify any linear relation between the spatial location of touch and pain perception. Rather, inspection of the data showed similar strong reductions in sensitivity for the 2 tactile locations closest to the nociceptive stimulation, but less modulation for the farthest tactile location. Dunnett’s post hoc tests comparing each L+T condition against L revealed a significant reduction of *d*’ when the touch was located at 1 (*P* = 0.021) or 5 cm (*P* = 0.009) either side from the laser pulse. The 2-way test for the largest laser-touch distance (9 cm) only showed a trend toward significance (*P* = 0.091).

#### 3.2.2 Response bias (C)

The first contrast showed that response bias was significantly modulated by the concurrent stimulation of Aβ fibres (*P* = 0.005). The contrast for the linear trend was also significant (*P* = 0.007). That is, the closer was the touch to the location of the laser pulse; the stronger was the negative response bias (Fig. 5b). Moreover, Dunnett’s post hoc tests revealed a significant bias in the L+T1 (*P* = 0.010) and L+T5 (*P* = 0.044) conditions, but not in the L+T9 condition (*P* = 0.751).

### 3.3 Discussion

Experiment 2 replicated in a new and larger group of participants the key findings of Experiment 1: that intrasegmental touch reduces the sensitivity to the laser energy, and induces a response bias toward lower pain levels. Moreover, this touch–pain interaction was shown to extend over a wider zone than Experiment 1.

We fitted a simple model of a linear relation between touch-laser distance and pain levels. We found a strong linear modulation of response bias. We also found a spatial relation between concurrent touch and sensitivity to laser energy, but this was not strictly linear in form.

Tactile stimuli separated by <2 cm are reported to spatially summate [27]. In our L+T5 and L+T9 conditions, the tactile stimuli were located, respectively, 5 and 9 cm either side of the laser pulse (the von Frey hairs were 10 and 18 cm apart). These spatial separations lie beyond the reported summation threshold [27]. Nevertheless, stimulus energy was balanced across the 3 distances tested, so spatial summation of the stimuli delivered 2 cm apart (ie, in condition L+T1) does not necessarily undermine our concept of a spatial gradient of tactile-nociceptive interaction. To further address this issue, we also considered whether there was a spatial gradient of touch–pain interaction just for those conditions beyond the plausible summation range (ie, L+T5 and L+T9 cm). We found a significant difference between the L+T5 and L+T9 cm conditions in spatial bias (paired *t*-test: *t*_(13) _= 2.35, *P* = 0.035), but not in sensitivity (*t*_(13) _= 1.55, *P* = 0.145). This additional analysis suggests a similar conclusion to the linear spatial trends computed from the whole dataset: a space-dependent bias in judgments about pain levels, and a nonspatial tactile impairment of sensitivity to pain levels.

## 4 General discussion

Experiments 1 and 2 both demonstrated that touch can inhibit pain. In addition to the reduction of subjective pain intensity reported previously (Experiment 1), we found that tonic touch reduces the perceptual sensitivity to concomitant laser pulses (Experiments 1 and 2). Aβ input also modulated participants’ bias to judge that the laser energy was “high” (Experiments 1 and 2). The effect of touch was so powerful that the probability of detecting any concurrent Aδ stimulus was dramatically reduced (Experiment 1). Further, touch appeared to influence stimulus quality of Aδ input, as judged by the choice of verbal descriptors. When detected, laser stimuli were not perceived as pinprick on 60%-70% of L+T trials (Experiment 1). Nevertheless, the latency to detect the Aδ stimulus was not significantly affected by concurrent Aβ stimulation (Experiment 1), and remained compatible with the conduction velocity of Aδ fibres [20].

Together, these results indicate that Aβ input has several effects on pain perception linked to the ascending Aδ pathway, for the following reasons. First, the sensory capacity of the ascending Aδ pathway may be decreased to the point where stimulation can become imperceptible. Second, when stimulation is perceived, *perceptual sensitivity* is sharply reduced. This corresponds to a functional loss of information about the stimulus energy within the Aδ ascending pathway. Third, the reduction of Aδ ascending information appears to alter stimulus quality, and to downregulate pain levels.

Previous studies often used prolonged tactile stimulation, typically reporting changes in pain perception (eg, [10,12,13,17,22,30]). Only one previous study combined spatiotemporally precise tactile input with nociceptive-selective laser stimuli [21]. However, this study did not find any tactile modulation of pain perception, and moreover, did not attempt to address spatial aspects of the touch–pain interaction. The tactile stimulation employed in that study [21] was much shorter than ours (1-ms transcutaneous electrical vs 1.5-second von Frey stimulation), and therefore not directly comparable.

### 4.1 Spatial dependency of touch–pain interactions

Our study provides clear evidence for a spatial organisation of touch–pain interactions within a single dermatome (Experiment 2). The spatial gradient of these interactions was clearly linear for measures of response bias. Specifically, participants were less likely to respond “high” as the tactile location approached the laser pulse location, irrespective of the actual level of laser energy. Response bias in SDT effectively measures the reported *level* of sensation, regardless of the actual stimulus energy. Indeed, our multivariate analysis (Experiment 1) confirmed that overall pain ratings and SDT measure of response bias were affected to very similar extents by concurrent touch. This general tendency to adjust pain levels downwards may be called tactile analgesia. Our results show, apparently for the first time in humans, that tactile analgesia has a clear intrasegmental spatial gradient. The spatial relation between the tactile location and the sensitivity to the laser energy is more complex, and less obviously linear.

### 4.2 Spinal mechanisms of touch–pain interactions

Interestingly, the spatial dependency of touch–pain interactions may be compatible with the neurophysiological properties of WDR neurons. WDR cells typically have a concentric RF arrangement, graded for sensitivity. The centre is excited by any cutaneous stimulus, while the surround is excited by nociceptive stimuli and inhibited by mechanical stimulation (for a review, see [14]), in a spatially dependent fashion [26]. At the periphery of the receptive field, only intense tactile stimuli can inhibit responses to a nociceptive stimulus. In contrast, nociceptive-specific (NS) responses are not affected in a spatially dependent fashion [25]. Furthermore, the RFs of WDR neurons are larger than those of NS neurons, and change depending on attention [4,5,9]. Translating these neurophysiological findings to our psychophysical study, it appears unlikely that the spatial gradient of modulation we observed in Experiment 2 is produced by changes in firing of NS neurons, while it is potentially compatible with the properties of WDR cells.

### 4.3 Supraspinal mechanisms of touch–pain interactions

Data from Experiment 1 provide some evidence consistent with an additional supraspinal mechanism. In monkeys, there is a significant correlation between the neural discharge of WDR neurons and the behavioural detection latency to noxious heat [16]. Detection latencies to laser stimuli are prolonged in humans during longer-lasting Aβ stimulation [continuous: 12; 4-6 s: 22]. In contrast, in our study, detection latencies were unaffected by concurrent touch, and remained consistent with conduction in the Aδ pathways. From this, we conclude that an afferent Aδ volley reached the brain with a similar latency with and without concurrent touch, despite any reduction of spinal neural activity. Moreover, this afferent volley sometimes failed to produce the pinprick quality generally associated with Aδ pain. These 2 findings suggest that some supraspinal mechanisms may have contributed to modulate the sensations evoked by the afferent volley.

Two main arguments are generally used to support the hypothesis of supraspinal mechanisms for pain relief by touch. First, segmental brushing has been found to reduce overall pain ratings and response biases, without modulating *d*’ measures of sensitivity to the nociceptive stimulation [22]. However, our Experiments 1 and 2 question this finding, since we observed strong decreases in sensitivity to the laser energy following naturalistic touch.

The second argument is based on the timing of nociceptive–tactile interactions. Inui et al. [10] found that somatosensory-evoked responses to nociceptive intraepidermal electrical stimulation are modulated by innocuous transcutaneous electrical stimulation, even when the nociceptive stimulus was given 60-20 ms earlier than the innocuous conditioning stimulus. Whether this finding generalises also to laser-evoked responses is still unclear. The linear effect of touch location on response bias (Experiment 2) is compatible with the existence of fine-grained somatotopic maps of Aδ input in primary somatosensory cortex, aligned with maps evoked by tactile input [18]. This does not, however, imply that the neural basis of the pain relief by touch is entirely cortical.

### 4.4 Role of attention

Could pain relief by touch simply reflect a distraction, or shift in attention from the nociceptive to the innocuous stimulus? We think this is unlikely. Our tactile stimuli were delivered as pairs bracketing the laser pulse location, so that the centroid locations of tactile and nociceptive stimuli were identical. Moreover, spatial attentional mechanisms would suggest that larger shifts in spatial attention away from the laser pulse location should produce greater reductions in pain processing. In fact, we found a highly significant effect in the opposite direction, at least for response bias.

Nor can our effects be explained by changes in salience or arousal due to tactile stimulation. We gave an acoustic stimulus (1.5 seconds before the laser onset) on every trial, in both L and L+T conditions. Thus, temporal expectancy for the laser stimulus was balanced across conditions, and was independent of both the presence and the location of touch.

Finally, we allowed an interval of 0.75 second between tactile onset and laser stimulation. This interval is an order of magnitude greater than those associated with forward masking and somatosensory imperception [3].

### 4.5 Benefits of an SDT approach

Many previous studies used signal detection methods to test pain detection (“Does the stimulus elicit pricking pain, yes or no?”) [24]. Our use, in contrast, focuses on the quality of information processing *within* the ascending Aδ pathway, which may be more relevant for understanding *how* tactile input influences the functional activation of Aδ circuits.

The most sensitive measure for detecting tactile modulation of pain in our study was SDT response bias, closely followed by ratings of pain intensity. The SDT measure of bias has indeed many psychometrically desirable properties. It has an intuitive correspondence with the concept of pain level or pain intensity. It does not require defining a specific quality of sensation, such as “first pain” or “pricking pain.” Unlike rating scales, it does not require a concept of stimuli that are not in fact presented (eg, “the worst pain imaginable”) used to anchor the extremes of a pain scale. In fact, the only psychometric requirement is that participants be capable of arranging a set of successive sensations according to an ordinal scale of magnitude. However, a disadvantage of this measure is that it requires the participants to be able to discriminate between 2 levels of stimulation, and to tolerate the more intense of the 2 stimuli.

### 4.6 Conclusions

Our study shows convergent evidence that touch induces analgesia in a spatially dependent fashion. That “touch inhibits pain” has been a central tenet of pain research for half a century, since the precursors of gate theory [19,28]. Surprisingly, this appears to be the first report studying the spatial organisation of intrasegmental touch–pain interactions in humans. Our results may be consistent with the neurophysiological characteristics of spinal multimodal neurons. However, we also show fine spatial and qualitative features of tactile modulation that may depend on additional, supraspinal mechanisms. The implication for pain relief by touch is that the spatial position of the Aβ stimulus matters, even within a segment.

## Conflict of interest statement

The authors declare no competing financial interests.

## Figures and Tables

**Table 1:** Standardised weighting coefficients from linear discriminant analysis (Experiment 1).

Response bias (*C*)	3.691
Numerical pain ratings	3.328
Numerical difference in pain ratings (medium vs high)	1.902
Sensitivity (*d’*)	0.717

## References

[1] Baumgartner U., Cruccu G., Iannetti G.D., Treede R.D. (2005). Laser guns and hot plates. PAIN®.

[2] Beissner F., Brandau A., Henke C., Felden L., Baumgärtner U., Treede R.-D., Oertel B.G., Lötsch J. (2010). Quick discrimination of A(delta) and C fiber mediated pain based on three verbal descriptors. PLoS One.

[3] Blankenburg F., Taskin B., Ruben J., Moosmann M., Ritter P., Curio G., Villringer A. (2003). Imperceptible stimuli and sensory processing impediment. Science.

[4] Dubner R., Hoffman D.S., Hayes R.L. (1981). Neuronal activity in medullary dorsal horn of awake monkeys trained in a thermal discrimination task. III. Task-related responses and their functional role. J Neurophysiol.

[5] Hayes R.L., Dubner R., Hoffman D.S. (1981). Neuronal activity in medullary dorsal horn of awake monkeys trained in a thermal discrimination task. II. Behavioral modulation of responses to thermal and mechanical stimuli. J Neurophysiol.

[6] Hays W. (1994). Statistics.

[7] Higgins J.D., Tursky B., Schwartz G.E. (1971). Shock-elicited pain and its reduction by concurrent tactile stimulation. Science.

[8] Hillman P., Wall P.D. (1969). Inhibitory and excitatory factors influencing the receptive fields of lamina 5 spinal cord cells. Exp Brain Res.

[9] Hoffman D.S., Dubner R., Hayes R.L., Medlin T.P. (1981). Neuronal activity in medullary dorsal horn of awake monkeys trained in a thermal discrimination task. I. Responses to innocuous and noxious thermal stimuli. J Neurophysiol.

[10] Inui K., Tsuji T., Kakigi R. (2006). Temporal analysis of cortical mechanisms for pain relief by tactile stimuli in humans. Cereb Cortex.

[11] Johansson R.S., Vallbo A.B., Westling G. (1980). Thresholds of mechanosensitive afferents in the human hand as measured with von Frey hairs. Brain Res.

[12] Kakigi R., Shibasaki H. (1992). Mechanisms of pain relief by vibration and movement. J Neurol Neurosur Psychiatry.

[13] Kakigi R., Watanabe S. (1996). Pain relief by various kinds of interference stimulation applied to the peripheral skin in humans: pain-related brain potentials following CO_2_ laser stimulation. J Peripher Nerv Syst.

[14] Le Bars D. (2002). The whole body receptive field of dorsal horn multireceptive neurones. Brain Res Brain Res Rev.

[15] Macmillan N.A. (1991). Signal detection theory: a user’s guide.

[16] Maixner W., Dubner R., Bushnell M.C., Kenshalo D.R., Oliveras J.L. (1986). Wide-dynamic-range dorsal horn neurons participate in the encoding process by which monkeys perceive the intensity of noxious heat stimuli. Brain Res.

[17] Malow R.M., Dougher M.J. (1979). A signal detection analysis of the effects of transcutaneous stimulation on pain. Psychosom Med.

[18] Mancini F., Haggard P., Iannetti G.D., Longo M.R., Sereno M.I. (2012). Fine-grained nociceptive maps in primary somatosensory cortex. J Neurosci.

[19] Melzack R., Wall P.D. (1965). Pain mechanisms: a new theory. Science.

[20] Mouraux A., Guerit J.M., Plaghki L. (2003). Non-phase locked electroencephalogram (EEG) responses to CO2 laser skin stimulations may reflect central interactions between A partial partial differential- and C-fibre afferent volleys. Clin Neurophysiol.

[21] Mouraux A., Plaghki L. (2007). Cortical interactions and integration of nociceptive and non-nociceptive somatosensory inputs in humans. Neuroscience.

[22] Nahra H., Plaghki L. (2003). Modulation of perception and neurophysiological correlates of brief CO_2_ laser stimuli in humans using concurrent large fiber stimulation. Somatosens Mot Res.

[23] Pantaleo T., Duranti R., Bellini F. (1986). Effects of vibratory stimulation on muscular pain threshold and blink response in human subjects. PAIN®.

[24] Rollman G.B. (1977). Signal detection theory measurement of pain: a review and critique. PAIN®.

[25] Salter M.W., Henry J.L. (1990). Differential responses of nociceptive vs. non-nociceptive spinal dorsal horn neurones to cutaneously applied vibration in the cat. PAIN®.

[26] Salter M.W., Henry J.L. (1990). Physiological characteristics of responses of wide dynamic range spinal neurones to cutaneously applied vibration in the cat. Brain Res.

[27] Von Bekesy G. (1958). Funnelling in the nervous system and its role in loudness and sensation intensity on the skin. J Acoust Soc Am.

[28] Wall P.D. (1996). Comments after 30 years of the Gate Control Theory. Pain Forum.

[29] Watanabe I., Svensson P., Arendt-Nielsen L. (1999). Influence of segmental and extra-segmental conditioning, stimuli on cortical potentials evoked by painful electrical stimulation. Somatosens Mot Res.

[30] Yarnitsky D., Kunin M., Brik R., Sprecher E. (1997). Vibration reduces thermal pain in adjacent dermatomes. PAIN®.

